# The Genetic Basis of the First Patient with Wiedemann–Rautenstrauch Syndrome in the Russian Federation

**DOI:** 10.3390/genes15020180

**Published:** 2024-01-29

**Authors:** Valeriia A. Kovalskaia, Anastasiia L. Kungurtseva, Fatima M. Bostanova, Peter A. Vasiliev, Vyacheslav Y. Tabakov, Mariia D. Orlova, Inna S. Povolotskaya, Olga G. Novoselova, Roman A. Bikanov, Mariia A. Akhyamova, Yulia V. Tikhonovich, Anastasiia V. Popovich, Alisa V. Vitebskaya, Elena L. Dadali, Oxana P. Ryzhkova

**Affiliations:** 1 Research Centre for Medical Genetics, 115522 Moscow, Russia; mikhailova.v.a@mail.ru (V.A.K.);; 2 Pediatric Endocrinology Department, I.M. Sechenov First Moscow State Medical University, 119991 Moscow, Russia; kungurtseva.al@mail.ru (A.L.K.);; 3 JSC “First Genetics Laboratory”, 111123 Moscow, Russia

**Keywords:** Wiedemann–Rautenstrauch syndrome, neonatal progeroid syndrome, *POLR3A*, mRNA analysis, WES

## Abstract

Bi-allelic pathogenic variations within *POLR3A* have been associated with a spectrum of hereditary disorders. Among these, a less frequently observed condition is Wiedemann–Rautenstrauch syndrome (WRS), also known as neonatal progeroid syndrome. This syndrome typically manifests neonatally and is characterized by growth retardation, evident generalized lipodystrophy with distinctively localized fat accumulations, sparse scalp hair, and atypical facial features. Our objective was to elucidate the underlying molecular mechanisms of Wiedemann–Rautenstrauch syndrome (WRS). In this study, we present a clinical case of a 7-year-old female patient diagnosed with WRS. Utilizing whole-exome sequencing (WES), we identified a novel missense variant c.3677T>C (p.Leu1226Pro) in the *POLR3A* gene (NM_007055.4) alongside two *cis* intronic variants c.1909+22G>A and c.3337-11T>C. Via the analysis of mRNA derived from fibroblasts, we reconfirmed the splicing-affecting nature of the c.3337-11T>C variant. Furthermore, our investigation led to the reclassification of the c.3677T>C (p.Leu1226Pro) variant as a likely pathogenic variant. Therefore, this is the first case demonstrating the molecular genetics of a patient with Wiedemann–Rautenstrauch syndrome from the Russian Federation. A limited number of clinical cases have been documented until this moment; therefore, broadening the linkage between phenotype and molecular changes in the *POLR3A* gene will significantly contribute to the comprehensive understanding of the molecular basis of POLR3A-related disorders.

## 1. Introduction

Sequence variations in *POLR3A* are characterized by high phenotypic heterogeneity, with manifestations ranging from neonatal progeroid syndrome and severe childhood-onset hypomyelinating leukodystrophy with hypogonadotropic hypogonadism to adult-onset gait disorders with spastic paraplegia and cerebellar ataxia [1,2,3]. To date, the less investigated disorder is a neonatal progeroid syndrome, also known as Wiedemann–Rautenstrauch syndrome, with a prevalence of less than 1:1,000,000 [4]. It is an autosomal recessive inherited neonatal progeroid condition characterized by prenatal and early postnatal growth retardation, marked general lipodystrophy with distinctive local accumulations of fat, sparse scalp hair, and atypical facial features. *POLR3A* encodes the largest subunit of the DNA-dependent RNA polymerase III, a polymerase that transcribes genes coding small RNAs, such as 5S rRNA and tRNAs. Initially, it was postulated that only truncating variants in *POLR3A* may result in Wiedemann–Rautenstrauch syndrome [5]; however, a set of missense mutations has already been described [6].

Here, we present a clinical case of a 7-year-old female patient with Wiedemann–Rautenstrauch syndrome carrying a novel missense variant c.3677T>C (p.Leu1226Pro) in the *POLR3A* gene (NM_007055.4) alongside two *cis* intronic variants c.1909+22G>A and c.3337-11T>C. Here, we reconfirmed the splicing-affecting nature of the c.3337-11T>C variant and classified a new missense c.3677T>C (p.Leu1226Pro) variant as a likely pathogenic one. Therefore, this is the first case demonstrating the molecular genetics of a patient with Wiedemann–Rautenstrauch syndrome from the Russian Federation.

## 2. Subject and Methods

### 2.1. Clinical Evaluation

**Figure 1:**
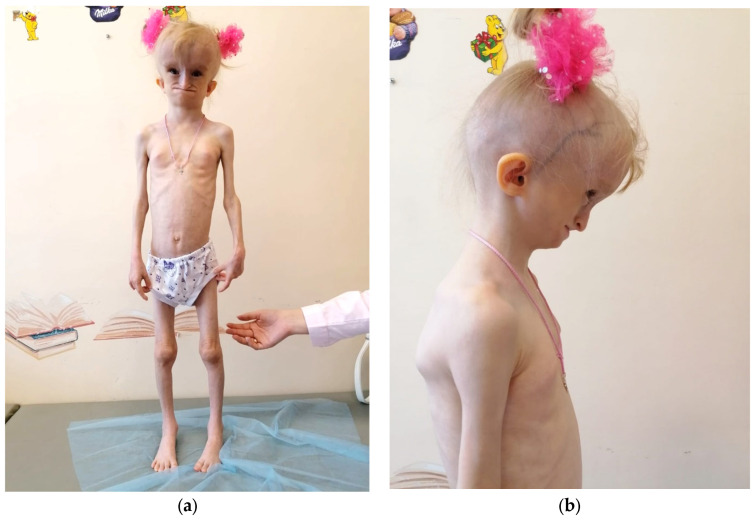
(**a**,**b**): Phenotypic features of an affected individual at age 7 years. Images show the distinctive progeroid facial features, including a general lipodystrophy with distinctive local accumulations of fat, a hydrocephalic cranium, a prominent venous network, pronounced massive frontal tubercles, a triangular face with midface hypoplasia, sparse eyebrows and eyelashes, a scalp hypotrichosis, low-set ears, a beak-shaped nose, a short philtrum, deep-set eyes, and a wide mouth.

**Figure 2:**
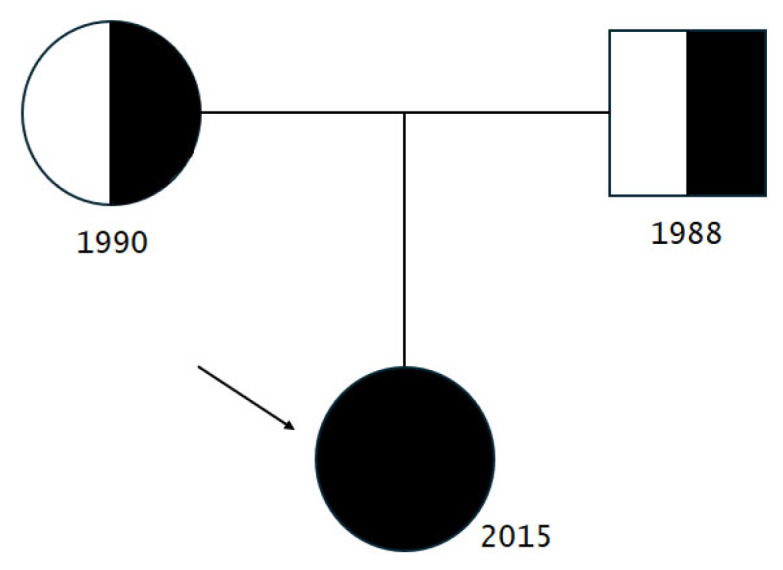
A pedigree of the affected subject.

The proband, a 7-year-old female Russian patient (Figure 1), underwent a comprehensive examination conducted by a pediatric endocrinologist at Sechenov University Hospital in the presence of her parents. Neonatal progeroid syndrome was suspected, so it prompted the necessity of subsequent evaluation by a clinical geneticist at the Research Centre for Medical Genetics. The marriage of the proband’s parents was non-consanguineous (Figure 2). Informed consent was obtained from both the proband and her parents for the performance of genetic tests and their anonymous participation in scientific research. Blood samples were collected from the family members for subsequent analysis, while the proband underwent a skin biopsy to establish a fibroblast cell culture as well.

### 2.2. Molecular Tests

Upon reception at the laboratory, the blood samples were used for DNA extraction utilizing the GeneJet Genomic DNA Purification Kit (Thermo Fisher Scientific, Waltham, MA, USA). Subsequent enrichment procedures were conducted using the SureSelect All Exon v7 kit (Agilent, Santa Clara, CA, USA). Whole-exome sequencing was performed using the MGISEQ-2000 platform. The acquired data underwent analysis via a tailored bioinformatics algorithm. An initial quality evaluation of the raw paired-end reads was conducted using the FastQC algorithm. The reads were aligned to the human reference genome assembly GRCh38 utilizing the bwa-mem2 software package (version 2). The identification of single nucleotide genetic variants, as well as short insertions and deletions, was carried out employing the strelka2 program. The obtained data achieved an average coverage depth of 152×, with 97.2% of targets having at least ×10 coverage and 96.0% of targets offering at least ×20 coverage. The annotation of identified genetic variants was executed utilizing the ENSEMBL-VEP program. Subsequently, the genetic variants were classified in accordance with the guidelines provided by the American College of Medical Genetics and Genomics (ACMG).

**Figure 3:**
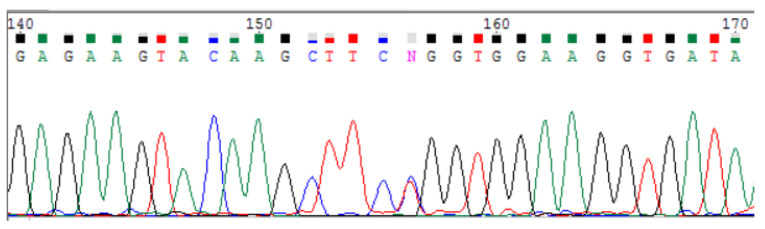
Sanger sequencing of the POLR3A (NM_007055.4) showing compound-heterozygous variant c.3677T>C (p.Leu1226Pro) inherited from the father.

**Figure 4:**
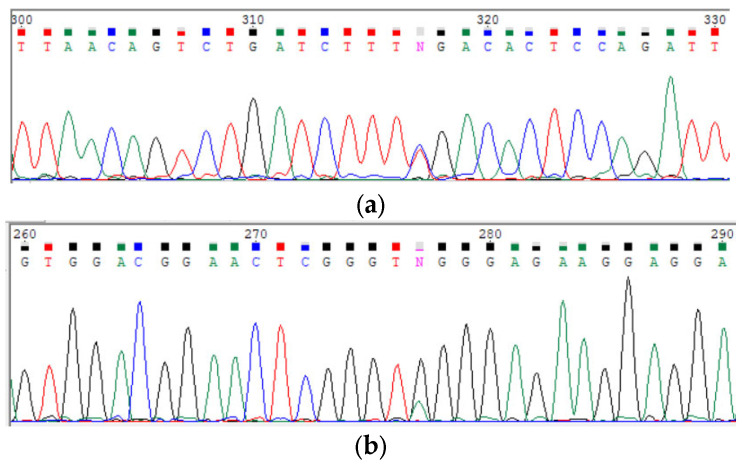
Sanger sequencing of the POLR3A (NM_007055.4) showing heterozygous variants c.3337-11T>C (**a**) and c.1909+22G>A (**b**) inherited from the mother, which comprises a complex allele.

**Figure 5:**
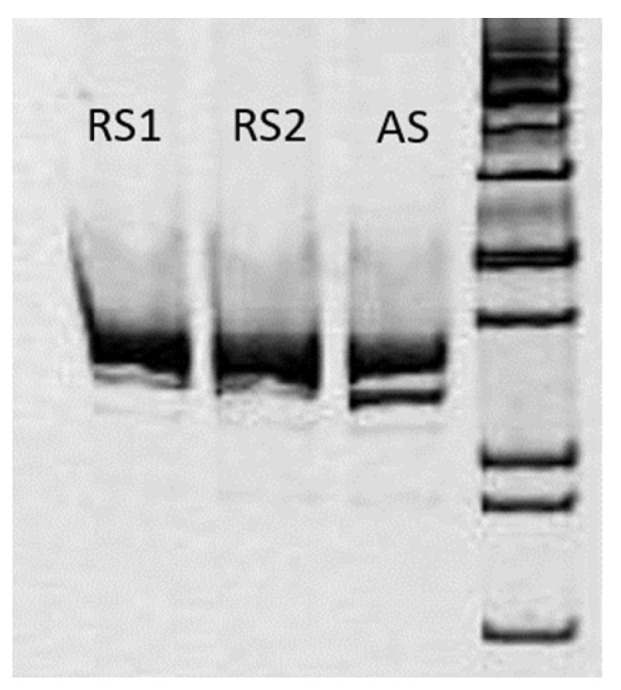
cDNA amplicons of the patient (AS) and healthy controls (RS1 and RS2) showed two different-sized PCR products in the patient sample. The upper product was revealed to belong to the wt transcript, and the lower band corresponds with the transcript with the 26th exon skipped, resulting in in-frame deletion, p.I1113_E1143del.

**Figure 6:**
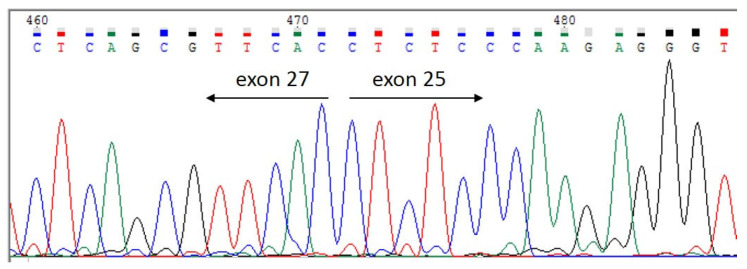
Sanger sequencing of the lower PCR product of the patient, demonstrating 26th exon skipping of the *POLR3A* gene.

**Figure 7:**
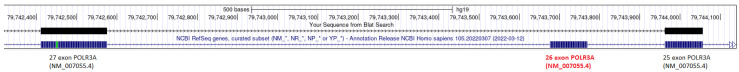
The patient’s lower band sequence alignment (USCS genomic browser) demonstrating 26 *POLR3A* exon skipping.

In order to validate *POLR3A* variants and confirm the trans-position of c.3677T>C (p.Leu1226Pro) and a complex allele c.[3337-11T>C; 1909+22G>A], Sanger sequencing for the proband and her parents was carried out (segregation analysis) (Figure 3 and Figure 4). The c.3337-11T>C splicing alteration and no splicing effect for c.3677T>C was confirmed via mRNA analysis (Figure 5, Figure 6 and Figure 7). Fibroblasts were collected from the proband via a forearm skin biopsy in accordance with current standards. The obtained fibroblasts were cultured at 37 °C with 5% CO_2_ in a DMEM medium supplemented with 15% FBS. Subsequently, primary cell cultures were established, and total RNA was extracted from the cultured fibroblasts using the QIAzol^®^ Lysis Reagent (Qiagen, Hilden, Germany). The quality of the extracted RNA was assessed via agarose gel electrophoresis and the 260/280 ratio, which was determined spectrophotometrically during RNA concentration measurement. Reverse transcription of the RNA samples was carried out using the QuantiTest^®^ Reverse Transcription Kit (Qiagen, Hilden, Germany). Standard PCR for cDNA samples of the proband and non-affected subjects was carried out using primers flanking exons 25 and 30 of the POLR3A gene (GCTTCCAAGGCCATCAGCAC and GGCCAGGCCAAACCTAGTGA). The amplicon length was initially calculated to be 700 bp.

## 3. Results

### 3.1. Clinical Findings

A female infant was born to non-consanguineous healthy parents. She presented profound intrauterine growth retardation, which became evident from the 24th week of gestation. The delivery was at 37 weeks of gestation via cesarean section, and right after birth, a weight deficit (1840 g) was revealed while body length was within normal limits (46 cm). The newborn exhibited a lack of subcutaneous fat, a hydrocephalic shape of the cranium, and was characterized by a prominent venous network across all regions, micrognathia of the lower jaw, and a neonate tooth (one upper incisor) spontaneously falling out on the 2nd day of life. During medical assessments in the first year of life, Arnold–Chiari malformation type 1, mixed hydrocephalus, and an occipital bone anomaly presenting as a defect in the posterior sections of the foramen magnum were revealed. Subsequent assessments identified severe low weight and global developmental delay (physical, motor, and speech).

At her current age of 7.5 years, the patient continues to exhibit severe growth retardation (height: 103 cm, SDS −3.41; growth rate: 3.64 cm/year, SDS −2.47) and profound body weight deficiency (weight: 10.35 kg, BMI: 9.71 kg/m^2^, SDS −6.20). Physical examination reveals distinctive progeroid facial features (Figure 1), including a hydrocephalic cranial shape marked by a prominent venous network, pronounced massive frontal tubercles, a triangular face with midface hypoplasia, sparse eyebrows and eyelashes, low-set ears, a beak-shaped nose, a short philtrum, deep-set eyes, a wide mouth, tooth agenesis, and an elongated tongue. Other notable physical findings include scalp hypotrichosis, a transverse palm crease on the right hand, a general lipodystrophy with distinctive local accumulations of fat (neck, external genitalia, coccygeal region, and feet), as well as hip joint and interphalangeal joints contractures.

Further systemic examinations revealed ophthalmological findings (retinal angiopathy, astigmatism, entropion, and an opacity of the left eye cornea), neurological complications (a congenital osteo-neural malformation of the craniocervical junction, an Arnold–Chiari malformation type 1, spina bifida C1, and dysarthria), orthopedic issues (scoliosis, hands, and knees flexion joints, and osteoporosis), and gastroenterological findings (reactive pancreas changes, ileal lymphofollicular hyperplasia, and steatohepatosis). The clinical findings are presented in a structured manner in Table 1.

### 3.2. Molecular Tests Results

#### 3.2.1. Whole-Exome Sequencing (WES)

**Figure 8:**
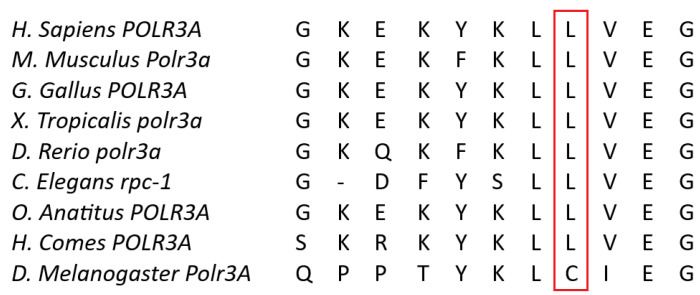
Interspecies POLR3A sequence comparisons demonstrating that the 1226 POLR3A amino acid is highly conserved.

**Figure 9:**
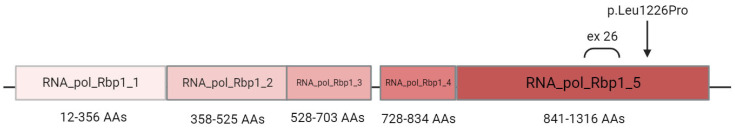
POLR3A human protein domains and the localization of the detected *POLR3A* gene variants.

**Figure 10:**
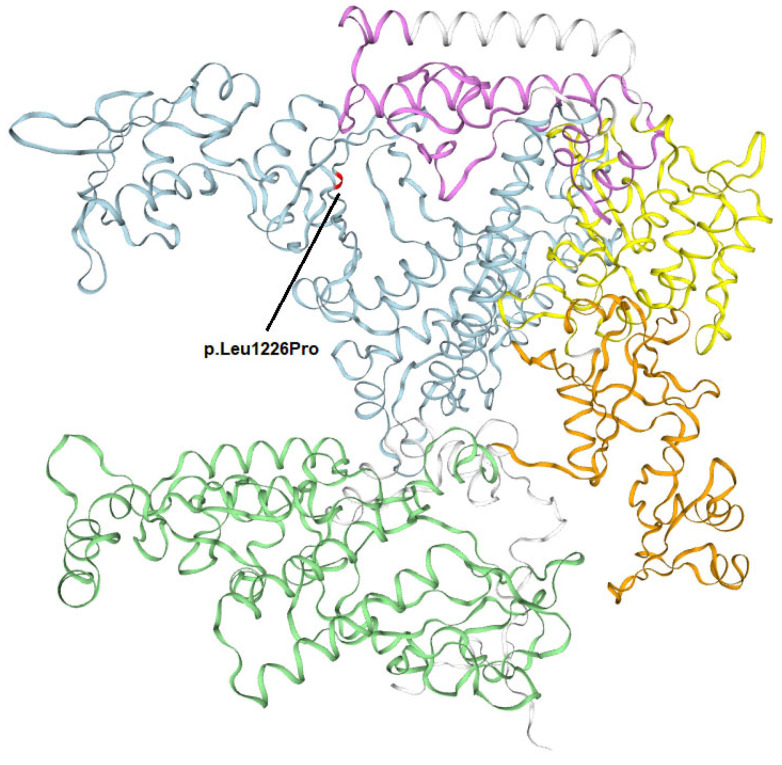
A three-dimensional modeling of the novel missense variant (p.Leu1226Pro) observed in this study.

Whole-exome sequencing (WES) revealed a previously unknown heterozygous missense variant in the *POLR3A* gene, NM_007055.4: c.3677T>C (p.Leu1226Pro), and two previously described heterozygous variants, c.3337-11T>C and c.1909+22G>A, which were reported several times to be in a complex allele [6]. Both c.3677T>C (p.Leu1226Pro) and c.3337-11T>C variants are present in the gnomAD database (v4) with low allele frequency, and the prevalence of c.1909+22G>A is 0.2409% (with 10 homozygous patients reported). PrimateAI, SIFT, Mutation assessor, and PolyPhen-2 predicted the c.3677T>C (p.Leu1226Pro) variant to be deleterious or probably damaging; no benign predictions were obtained. Bioinformatics analysis using SpliceAI predicted donor loss (Δ score 1.00) in −103 bp and acceptor loss at −11 bp position (Δ score 0.99) for c.3337-11T>C and a slight splicing alteration for the c.3677T>C (p.Leu1226Pro) variant, the Δ score 0.47 in −8 bp for donor gain, and the Δ score 0.32 at the position 372 bp for donor loss. Subsequently, genetic variants were classified in accordance with the guidelines provided by the American College of Medical Genetics and Genomics (ACMG) and the Association for Molecular Pathology (AMP) [7]. According to these guidelines, c.3337-11T>C was classified as a likely pathogenic variant (PM2, PS3, and PP5), and c.3677T>C (p.Leu1226Pro) was classified as a likely pathogenic variant as well (PM2, PP3, PM3, and PP2); moreover, PP4 was additionally applied to c.3677T>C (p.Leu1226Pro) as the phenotype specificity for Wiedemann–Rautenstrauch syndrome is high. The 1226 amino acid position is highly conservative among species (Figure 8) and is considered to be a part of RNA_pol_Rpb1_5 domain (Figure 9 and Figure 10), as well as the amino acids coded by POLR3A exon 36, and it is required to form the central cleft or channel where the DNA is bound. The CADD Phred score for c.3677T>C (p.Leu1226Pro) is 29.2 (RawScore is 4.258505), the gerp score is 5.99, and the Revel score is 0.892.

#### 3.2.2. Sanger Sequencing

Via Sanger sequencing, it was proved that c.3677T>C (p.Leu1226Pro) and c.[3337-11T>C;1909+22G>A] POLR3A variants are in the compound-heterozygous state, as they were confirmed to be inherited from the parents (Figure 3 and Figure 4).

#### 3.2.3. mRNA Analysis

The c.3337-11T>C variant creates a cryptic splice site, causing exon 26 skipping, leading to the in-frame deletion of 31 amino acids. (Figure 5, Figure 6 and Figure 7).

## 4. Discussion

Disease-causing variants in the POLR3A gene were initially identified among individuals diagnosed with hypomyelinating leukodystrophy, also known as 4H leukodystrophy (Hypomyelination, Hypodontia, Hypogonadotropic Hypogonadism) and leading to manifestations such as gait ataxia, motor delay, dystonia, and additional complications [8]. Later, the associations between POLR3A gene pathogenic variants and neonatal progeroid syndrome, as well as spastic paraplegia and ataxia syndrome, were established. Nonetheless, a comprehensive understanding of the precise phenotype resulting from POLR3A pathogenic variants remains elusive, and accurate prediction of their impact on health remains a challenge.

One speculates that only bi-allelic splicing or truncating variants are associated with the WRS phenotype, and the genotypes with bi-allelic missense or missense changes in trans with splicing or truncating variants are associated with the distinct phenotype of hypomyelinating leukodystrophy [3]. However, an earlier study demonstrates that missense mutations in compound-heterozygous states with LOF variants may also result in the Wiedemann–Rautenstrauch phenotype. Paolacci S. [6] reported patients with WRS with the missense variants, which locate in the RNA_pol_Rpb1_5 domain only (p.Gly903Arg, p.Arg1069Gln, p.Lys1131Arg, p.Asp1292Asn, and p.Gly1335Arg), as well as a missense mutation described in this study does. However, 5th domain missense variants (Asp905Asn, p.Glu937Lys, and p.Leu1129Leu) in combination with splice-variants (c.1771-6C>G or c.1771-7C>G) may also result in extrapyramidal movement disorder with striatal involvement [9]. At the same time, hypomyelinating leukodystrophy is predominantly driven by missense mutations in 1–4 protein domains [2,10]; nevertheless, the exceptions are also described [11]. It is also worth mentioning that c.1909+22G>A in a compound-heterozygous state with any type of POLR3A variant, regardless of the protein domain, results in spastic ataxia or 4H leukodystrophy [2,12,13,14]. It is considered to be a hypomorphic variant, as its gnomAD allele frequency reaches 0,24% (with 10 homozygotes) [15]. It may cause hereditary spastic paraplegia and cerebellar ataxia with an adolescent- or adult-onset [12,16] but may also drive 4H in a newborn (1.5 months) [14]. In WRS syndrome, c.1909+22G>A is observed only in a complex allele with c.3337-11T>C and never appears alone. Paolacci S. [6] postulated a modest impact on transcript processing of the c.1909+22G>A variant, which alone is insufficient to diminish the functionality of the transcripts for a recessive allele. However, when cooccurred with the c.3337–11T>C on the same allele, there is an escalation in the aberrant splicing of POLR3A transcripts to a degree significant enough to cause the disease when coupled with a loss-of-function mutation as a second allele. In this study, we demonstrate that the missense alteration c.3677T>C (p.Leu1226Pro) as a second allele is also sufficient in driving Wiedemann–Rautenstrauch syndrome.

Currently, approximately 19 cases of Wiedemann–Rautenstrauch syndrome have been documented globally. The clinical manifestations observed in patients with WRS exhibit substantial alikeness, as they are characterized by intrauterine growth restriction (IUGR), reduced birth weight, progeroid features, a broad forehead, pronounced scalp veins, a triangular face, along with diminished adipose tissue and sparse hair. Inconsistent manifestations encompass skeletal and endocrine abnormalities, hearing impairment, ocular findings, as well as tremors, ataxia, and intellectual disability. The clinical characterization of the patient described in this study majorly corresponds with previously described patients; however, additional signs are also observed (Table 1).

## 5. Conclusions

We conclude that the type of mutation and protein domain position in *POLR3A* are not the only factors that determine the POLR3A-related phenotype, and it is apparently influenced by other genetic or epigenetic factors as well. The detailed description of the molecular alterations in patients with POLR3A-related disorders, especially with Wiedemann–Rautenstrauch syndrome may significantly contribute to understanding this issue.

## Figures and Tables

**Table 1:** Clinical characterization of the patient presented in this study.

Feature	Characterization
Current Age	7.5 years
Sex	Female
Pregnancy and delivery	Profound IUGR from the 24th week. Delivery at 37 weeks of gestation via cesarean section
Birth parameters	A weight deficit (1840 g), body length of 46 cm, and an evident lack of subcutaneous fat
Craniofacial features	Progeroid facial features, a hydrocephalic shape of the cranium with a prominent venous network across all regions, micrognathia of the lower jaw, and a defect in the posterior sections of the foramen magnum
Dental abnormalities	A neonate tooth (one upper incisor) spontaneously falling out on the 2nd day of life.
Postnatal growth	Severe growth retardation (height: 103 cm, SDS: 3.41; growth rate: 3.64 cm/year, SDS: 2.47) and profound body weight deficiency (weight: 10.35 kg, BMI: 9.71 kg/m^2^, SDS: 6.20).
Fat tissue distribution	A general lipodystrophy with distinctive local accumulations of fat (neck, external genitalia, coccygeal region, and feet)
Skin findings	A transverse palm crease on the right hand
Bone and joint findings	Hip joints and interphalangeal joints contractures, scoliosis, hands and knees joint flexion, and osteoporosis
Neurologic and developmental abnormalities	A congenital osteo-neural malformation of the craniocervical junction, an Arnold–Chiari malformation type 1, spina bifida C1, and dysarthria. No intellectual disability
Vision and hearing	A retinal angiopathy, an astigmatism, an entropion, and an opacity of the left eye cornea
Additional findings	Reactive pancreas changes, ileal lymphofollicular hyperplasia, and steatohepatosis
Family history	Non-consanguineous healthy Russian parents and no siblings
*POLR3A* variants	c.[3677T>C];[3337-11T>C;1909+22G>A]

## Data Availability

All data may be presented upon request. The data are not publicly available due to privacy reason.
